# Investigation on Dynamic Calibration for an Optical-Fiber Solids Concentration Probe in Gas-Solid Two-Phase Flows

**DOI:** 10.3390/s130709201

**Published:** 2013-07-17

**Authors:** Guiling Xu, Cai Liang, Xiaoping Chen, Daoyin Liu, Pan Xu, Liu Shen, Changsui Zhao

**Affiliations:** Key Laboratory of Energy Thermal Conversion and Control of Ministry of Education, School of Energy and Environment, Southeast University, Nanjing 210096, China; E-Mails: guilingxu@seu.edu.cn (G.X.); xpchen@seu.edu.cn (X.C.); dyliu@seu.edu.cn (D.L.); 230099066@seu.edu.cn (P.X.); 220120371@seu.edu.cn (L.S.); cszhao@seu.edu.cn (C.Z.)

**Keywords:** optical-fiber probe, solids concentration, calibration method, gas-solid two-phase flows

## Abstract

This paper presents a review and analysis of the research that has been carried out on dynamic calibration for optical-fiber solids concentration probes. An introduction to the optical-fiber solids concentration probe was given. Different calibration methods of optical-fiber solids concentration probes reported in the literature were reviewed. In addition, a reflection-type optical-fiber solids concentration probe was uniquely calibrated at nearly full range of the solids concentration from 0 to packed bed concentration. The effects of particle properties (particle size, sphericity and color) on the calibration results were comprehensively investigated. The results show that the output voltage has a tendency to increase with the decreasing particle size, and the effect of particle color on calibration result is more predominant than that of sphericity.

## 1. Introduction

**Figure 1.**
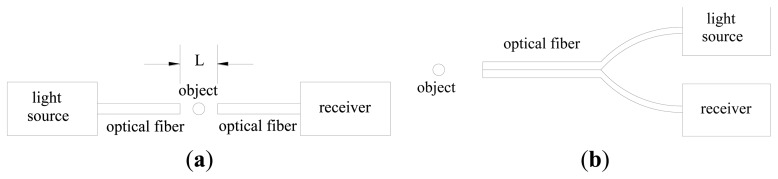
Two different types of optic fiber probes [19] (**a**) transmission-type; (**b**) reflection-type.

The measurements of solids concentration are essential to understand the gas-solid flow behavior in fluidized beds, blow tanks, pneumatic conveying lines, and other multiphase flow systems. A detailed knowledge of solids concentration profile is critical to the accurate design and valid modeling of these systems. Many techniques have been carried out for measuring solids concentration: X-ray or γ-ray absorption, laser Doppler anemometry, acoustics methods, capacitance probes, optical-fiber probes, and so on [1-7]. Optical-fiber probes have been widely used in recent years for the determination of velocity and concentration of particles in gas-solid flow systems [1-3,8-17], which have the advantages of high sensitivity, fast response, high signal-to-noise ratio, large dynamic range, small volume and light weight, fire and shock resistance and corrosion proof, freedom from disturbance by temperature, humidity, electrostatics and electromagnetic fields, and suitability for remote transmission and multi-channel detection. The application of optical-fiber probes to the solids concentration measurement is based on the principle that the particles in the fluid produce scattering of incident light [18]. There are two different arrangements of optical-fiber probes [19]: transmission-type probe and reflection-type probe, as shown in Figure 1.

As for the transmission-type probe, which is based on the forward scattering of particles against the incident light, the object to be measured is located between the two probe tips and the light input and output are coaxial. The effective measurement volume is dependent on the distance between the two probe tips *L*, diameter and numerical aperture of the probe. The output signal is independent of the chromaticness of particles, which means that a single white or black particle may produce the same output signals. As for the reflection-type probe, which is based on the back scattering of incident light by particles, it has only one tip, where the projecting and receiving fibers are intermingled or in rows, in parallel or crossed. The effective measurement volume is dependent on the diameter, numerical aperture, overlap region of the capture angles and the optic sensitivity of the photoelectric converter. The output signals depend on the chromaticness and reflectivity of the particles [18]. The transmission-type probe is restricted to relatively low solids concentration and considered to cause strong disturbance of the flow structure compared to the reflection-type probe [13,20]. The reflection-type probe may be used over the entire range of particle concentration, from extremely dilute flows to the fixed bed state [21]. Thus, more attention has been paid to the reflection-type optical-fiber probe.

**Figure 2.**
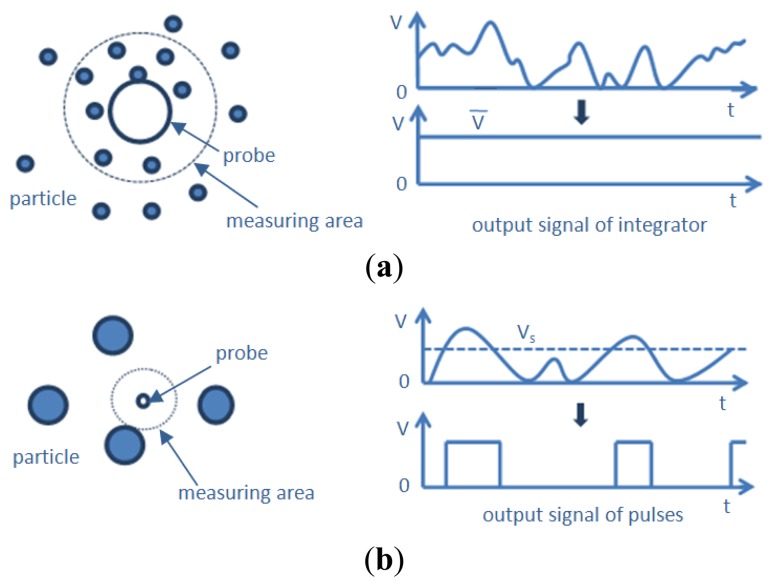
Two categories of reflection-type probe [19,22].

According to the ratio of particle diameter to fiber diameter, Matsuno *et al.* [19] classified the reflection-type probes into two categories. Figure 2a shows the optical-fiber probe with fiber diameter larger than particle diameter, the output signals are all generated by the reflected light from the particles existing within the measuring area. The integrated values of the output signal can be correlated with the particle concentration by any calibration method, and the instantaneous concentration can be obtained. Figure 2b shows the optical-fiber probe with fiber diameter smaller than the particle diameter. The output signals from the light receiver are converted into pulses at some threshold level *V_s_*, and the pulse count corresponds to the number of particles. The particle velocity must be known in order to convert pulses to concentration. Only the average concentration can be measured if the flow field fluctuates.

For the measurement of solids concentration, the emitted light reflected by the moving particles is magnified by a photo-multiplier and converted into voltage signals. The optical-fiber solids concentration probe should be calibrated to obtain a relationship between the output voltages and the solids concentration, which depends on the particle size distribution, concentration and optical properties of the solids and the surrounding fluid. The reliability of the measurements is strongly affected by the accuracy of the calibration method [9,16,21,23]. Calibration of a probe is finding a calibration curve with experiments and computations to convert the voltage time series into solids concentration time series [24]. The appropriate calibration method must be suitable for a wide range of solids concentrations from very low concentrations up to the solids concentrations of a packed bed [14]. However, it is difficult to generate a reference suspension of particles with a given concentration, and it is nearly impossible to produce a homogeneous gas-solid flow. Thus, calibration has become a problem.

The output signals of the reflection-type probe depend, to a large extent, on the aforementioned chromaticness of the particles, and the intensity of reflected light of white and black particles of the same diameter may differ by several times, and the roughness of particle surfaces may also have a similar influence [18]. Consequently, a calibration is required for each kind of particle. Very few studies have been carried out with regard to the effects of particle properties on the calibration curve of an optical-fiber solids concentration probe systematically and comprehensively, hindering the progress on the accurate calibration of the optical-fiber solids concentration probes.

The objective of this paper is to carry out investigation on dynamic calibration for an optical-fiber solids concentration probe in gas-solid two-phase flows. The remainder of this paper is organized as follows: a brief review of existing techniques for the calibration of optical-fiber solids concentration probes has been presented in Section 2. From the literature survey, it can be concluded that satisfactory calibration procedures are lacking in the literature and the calibration methods mainly focus on the low solids concentration. Thus, a calibration method, which is capable of calibrating the optical-fiber at nearly full range of the solids concentrations from 0 to packed bed concentration, is proposed. We combined the calibration method proposed by Hong *et al.* [23] with the calibration method used by Qi *et al.* [24]. The detailed experimental setup and calibration procedures are described in Section 3. With this combined method, the probe was uniquely calibrated with two kinds of powders. Finally, the calibration results are discussed and summarized in Section 4. The effects of particle properties on the calibration curve were also investigated.

## 2. Literature Survey

Both the transmission-type probe and the reflection-type probe needed to be calibrated for their measuring range before using them for the solids concentration measurement. Several techniques for the calibration of optical-fiber probes have been developed.

Matsuno *et al.* [19] calibrated the optical-fiber probe containing a pair of bundles of two small plastic optical fibers by employing the free-falling particles at their terminal velocities after having travelled a certain distance. The particles were poured through a vibrating sieve at a sufficient height to fall at a uniform velocity. The particle concentration was varied by changing the weight of particle on the sieve which was located at the top of the system and also by using sieves of different apertures. The probe was set at a height sufficiently below the sieve. The solids concentration was calculated with the following equation:

(1) $${ɛ}_{s}=\frac{\Delta W}{Au_{t}\Delta t}$$

where *ΔW* is the cumulative weight of particles on the cross-sectional area *A* within time *Δt*, and *u_t_* is the calculated terminal velocity of the particles. An approximately linear relationship between the output voltage and the solids concentration was obtained. This method is usable for measuring the solids concentration certainly with a maximum concentration of 0.001. The manual vibration of the sieve and the fouling of particles will produce some erroneous data. The method is not only unsuitable to the system in which particles have high velocities but also to fast fluidized beds where the clusters of particles are formed.

Cutolo *et al.* [15] calibrated his probe with an apparatus consisted of a solids feed hopper and a 41 mm i.d. and 1 m high Plexiglas pipe. The particles were charged into the hopper and fell downward through a series of nets which acted as a solids distributor. The solids flow rate was regulated by the number of nets and their meshes. Measurement was made throughout the pipe core at a distance from the axis from -15 mm to +15 mm. A smaller tube with 33 mm i.d. was coaxially positioned at the bottom of the high pipe, which allowed the separation of solids falling along the walls of the larger tube. A collecting vessel placed on a balance was located below the apparatus. The average solids concentration *e_s,v_* in the pipe of cross section *A* could be calculated with the following expression:

(2) $${ɛ}_{s,v}=\frac{\Delta W}{\rho AU({ɛ}_{s,v})\Delta t}$$

where *ΔW* is the weight of the particles collected during the time internal *Δt*, *ρ* is the particle density, *A* is the cross-section area of the pipe, *U* is the average falling velocity of the particles, and *U(ɛ_s,v_)* is a function involved of gas pressure and composition, particle size, concentration and weight. The relationship between the output voltages and solids concentration shows good linearity for the condition that the solids concentration is below 0.1. The maximum solids concentration achieved by using this method is 0.16.

Lischer and Louge [20] calibrated the optical-fiber probe used for measuring the particle volume fraction in dense suspensions against a quantitative capacitance probe. The material used was poured randomly along the probe assembly, which was mounted flush with the inside wall of a pipe that had a 15 cm i.d. They found that in a practical system, such regular calibration may be mandated by long-term variations of the average backscattered signal caused by subtle changes in the optical alignment or the quality of the fiber tip exposed to the particle suspension. However, the values from the optical-fiber probe and the capacitance probe didn't agree well.

Yamazaki *et al.* [25] carried out calibration experiments in a flat-bottomed cylindrical tank of 6.0 cm diameter. A known mass of solids was charged into a tank filled with water. After a steady state suspension was reached, the intensity of reflected light from the solids particles was measured by immersing the probe at various angular positions in the stirred tank. The light reflected from the solid particles in the solids-liquid mixtures had been measured in the range of solids concentration from 5% to 40% by volume. The axial concentration profiles at different radial positions agreed well, which implied that a homogeneous suspension of solids in the radial direction could be obtained by this method. It was found that measureable range for solids concentration was affected by particle species, particle diameter, particle shape, and particle color, variations in the refractive index of particles and continuous phase.

Zhou *et al.* [26] calibrated their optical-fiber solids concentration probe in two liquid-solid systems. For voidages less than 0.8, the calibration was carried out in a liquid-solid fluidized bed for the reason that particles were quite uniformly distributed in such a system. The solids concentration could be obtained with the following expression:

(3) $${ɛ}_{s}=\frac{H_{0}}{H}(1-{ɛ}_{0})$$

where *H_0_* is the height of a packed bed and *ε_0_* is the voidage of the packed bed. Concentration data were obtained by changing the fluid velocity to vary the bed height, *H*. For voidages larger than 0.8, the calibration was carried out in a beaker, where a certain volume of solid particles was put into and mixed with water of known volume. The liquid-solid mixture was stirred until the particles were uniformly distributed in the water. The calibration was very nearly linear over the entire solids concentration range obtained. Different concentrations were achieved by mixing different volumes of particles into water. However, it must be pointed out that water calibration may cause measurement errors while using the probe in a gas-solid flow due to the refraction index difference between liquid and gas. The index of refraction of a liquid is greater than that of a gas, and the probes should be calibrated in the same medium for which they will be utilized [27]. Furthermore, calibrations should also be performed accordingly for each kind powders or a certain kind of powders with different diameter, different color and particle shape based on the research of Yamazaki *et al.* [25].

Herbert *et al.* [21] calibrated a single-fiber optical reflection-type probe with a technique similar to that used by Cutolo *et al.* [15]. They established a stable downward flow of particles at a known velocity and in a column small enough so that a local measurement could yield a cross-sectional average value. The particles flowed from a fluidized bed feeder with a diameter of 12.5 cm, through an orifice in the center of the porous metal grid into a tube of square cross-section 8 × 8 mm, then fell 2.5 m into a collection pot. The particle flow rate from the fluidized bed could be kept stable by changing the orifice diameter. The average solids concentration could be calculated with the following expression:

(4) $${ɛ}_{s,v}=\frac{m_{p}}{u_{p}A\rho_{p}}$$

where *m_p_* is the particle mass flow rate, *u_p_* is the particle velocity measured with an optical-fiber velocity probe, *A* is the tube cross-sectional area, *ρ_p_* is the particle density. During the experiments, it was found that electrostatic forces caused fine particles to cling to the probe tip and obscured light transmission, which was very undesirable since the reflected light intensity was no longer a function of the particle concentration in front of the probe, but also related to the degree of coverage of the fiber which could not be known. To overcome the electrostatic force effect, a fine-particle additive Larostat 519 was mixed with the particles.

Hong *et al.* [23] developed a method for calibrating the optical-fiber probe using a polynomial regression to correlate the output signal with the solids volume concentration in the fluidized calibration vessel. Solid particles of mass *W_s_* were placed in the fluidized vessel made of transparent glass and then fluidized to the full vessel volume after closing the top end of the vessel with a filter screen. The overall volume concentration of solids within the full vessel could be calculated with the following expression:

(5) $${ɛ}_{s}=\frac{W_{s}}{\rho_{s}AH}$$

where *ρ_s_* is the solids density, *A* is the cross-sectional area of the vessel and *H* is the vessel height. They found that simply assuming a linear relationship between the output signal and solids concentration may cause a significant deviation from experiment in the range of concentration above 0.05. They also pointed out that a powder with very homogeneous fluidized behavior would have a more reliable calibration curve using their method. The disadvantage of this method was that the formation of bubbles in the bed during calibration which would cause some scatter of the calibrating points.

San José *et al.* [28] calibrated the optical-fiber probe used for voidage measurement of a conical spouted bed. For the spout zone, the calibration was carried out in a 60 mm i.d. column, where the probe had been introduced at a given level. The solid was fed by a hopper to the column. A linear relationship between the intensity of the reflected light and the volume fraction of the bed occupied by the particles, 1 − *ε*, was obtained. Bed voidage, *ε*, which was changed by adjusting the solid mass flow rate in the feed, Q, could be calculated with the following expression:

(6) $$Q=\rho Av_{z}(1-ɛ)$$

where *ρ* is the particle density, *A* is the column cross sectional area, *v_z_* is the velocity of the particles along the longitudinal direction at the probe zone, which can be calculated as follows:

(7) $$v_{z}=\frac{d_{e}}{\tau }$$

where *d_e_* is the effective distance between the two receiving fibers, *τ* is the delay time between the two signals. For the annular zone, the calibration consisted of measurement in moving beds, in a column of 60 mm i.d., using different particle sizes. Three kinds of different experiments were carried out: packed beds, beds loosened to the maximum and partially loosened beds. They found that the position of the probe in the bed did not affect the resulting calibration curve. This method has two disadvantages that the solids concentration along the cross-section of the downer column is mal-distribution and using the measurement of particle velocity with optical-fiber to evaluate solids concentration is not absolutely a good measurement technique.

Zhang *et al.* [27] proposed a back pressure control method to calibrate a multi-fiber optical reflection probe in a downer to obtain quantitatively precise solids concentration. The calibration apparatus was similar to that of Herbert *et al.* [21], and the significant improvement was adding a back pressure control system by sealing the bottom collection vessel. By using quick closing valves [1], the particles were trapped to determine various solids concentrations to compare with the data obtained with optical-fiber probe. The solids concentration could be obtained as large as 0.56 because the increase of the back pressure decreased the particle velocity and increased the solids concentration. Meanwhile, an iteration procedure was employed to modify the initial calibration curves. They also found that the probes were sensitive to minor variations of particle color and reflective properties. However, the flow was not entirely radial uniform even by using a vibrator to distribute the particles in the downer, which would cause calibration errors.

Johnsson *et al.* [29] carried out calibration experiments of an optical-fiber probe in a cold CFB riser, and compared the output signals with those obtained by an optical reference probe, which was calibrated with a guarded capacitance probe. The guarded capacitance probe was calibrated from measurements in a packed bed. They found that the reference probe and the optical-fiber probe gave similar response in amplitude and frequency with respect to variations in solids volume-fraction. The shape of the calibration function of the reference probe was also valid for the optical-fiber probe. The calibration and the reference measurement were carried out at ambient conditions, while the measurements were done at 850 °C with the assumption that the shape of the calibration function obtained under ambient condition was valid at elevated temperatures. They believed that it was a reasonable assumption because the shape of the calibration function depended on the optical properties of the probe and not on the temperature of the gas and particles.

Cui *et al.* [30] suggested a novel calibration method and correlation for different optical-fiber probes. They made a series of uniform mixture samples of FCC and amorphous transparent polystyrene by blending the two materials in a Brabender mixer at 200 ° C, shaped the samples into a cubic form in a size of 20 mm, and finally polished them to attain good optical properties. For different samples, the FCC particle concentration varied from 0 to that of the minimum fluidization state. The mixtures were used to simulate gas-solid flow systems with different solids concentrations, in which the transparent polystyrene was seen as air. The transparent polystyrene had refractive index higher than that of air. They found that the cross probe and parallel probes with glass window had similar calibration curves. The output voltages of the probe increased sharply with increasing solids concentration at low concentration but increased slowly at high solids concentration. This method is very interesting and novel. The probes were calibrated with a homogeneous dispersion of solids in the polymer-solid cubes with different and exact solids concentrations which is representative of different solids concentrations inside the gas-solid fluidized bed.

Rundqvist *et al.* [10] proposed an improvement in the design of a dual optical-fiber probe as well as a general calibration theory. They calibrated the probes in a small stirred tank filled with water, and a controlled mass of particles was added. The results of their calibration experiments showed some discrepancies relative to theoretical calibrations. A disproportional fraction of the particles were observed to reside close to the walls and the bottom of the calibrating vessel, which hindered the calibration experiments, leading to lower volume fractions than expected and offered a possible explanation to the above mentioned discrepancies. It was concluded that the calibration theory scaled well with probe size, particle size and particle volume fraction, although the exact shape of the calibration function could not be verified exactly.

Liu *et al.* [22,31] calibrated three-fiber optical probes with two units. The first was a dropping/trapping technique [32]. Particles fall into a collection vessel from an incipiently fluidized bed through a short tube located in the center of a punched plate distributor covered with fine wire mesh into a 12 mm i.d. tube. After a steady flow was obtained, two slide plates located 32 mm apart were closed quickly and simultaneously to trap the particles in a section of pipe where the probe inserted. By using different sizes of feeding tube and the flow rate of aeration air, different solids concentration could be obtained. The second was to obtain a water-FCC suspension in a well-stirred beaker. The probe with and without the quartz glass window was calibrated using both the two calibration units, and the results were compared with simulation predictions. The simulation results were in good agreement with the calibration results. They found that without the glass window, the calibration curves were highly nonlinear, which meant that the protective window could improve the linearity of the calibration curve. They also suggested that one should not directly apply calibrations obtained in liquids to calibrate probes for gas-solids systems.

Magnusson *et al.* [33] calibrated a dual fiber-optical probe based on the calibration theory proposed by Rundqvist *et al.* [10] in a circulating fluidized bed, for the reason that circulating fluidized beds provide a wide range of flow conditions and solids volume fractions. The particle volume fractions measured by the optical-fiber probe were compared to the pressure drop measured for a range of operation conditions. The relation between the pressure drop and the solids volume fraction could be expressed as follows:

(8) $$\Delta P={ɛ}_{s}(\rho_{s}-\rho_{g})gz+p_{\text{acc}}$$

where *ε_s_* is the solids volume fraction, *ρ_s_* and *ρ_g_* are the solids and gas phase densities, *g* is the acceleration due to gravity and *z* is the distance between the pressure measuring taps, *p_acc_* is the pressure drop due to acceleration of the particles, which may be neglected. They found that glare points on the particles and the beam length from the probe to the particle determines the curvature of the calibration curve. The values obtained with pressure drop measurements and the values of optical-fiber probe measurements showed poor agreement. The measurement volumes for the optical probe and the pressure drop are different from each other. Thus, application of this method for calibration of optical-fiber solids concentration probe is not satisfactory.

As can be seen from the literature review above, most of the researchers performed their calibration procedure and obtained specific kind of calibration curve, either linear or non-linear. All the calibration procedures were established by comparing the optical-fiber probe output signals to the concentration values obtained by the traditional methods for direct measurement of solids concentration, which could be mainly divided into three categories. The first is to use quick closing valves [1], with which the column to be studied can be positioned in sections of suitable length. The two valves were used to trap the solids within the desired section of the downer. After a certain period of time, the valves were closed simultaneously. The solids contained in the section can be collected and weighed, thus the solids concentration may be determined. This method was used by Zhang *et al.* [27] and Issangya *et al.* [32]. The second is to measure the pressure drop over a certain section of a riser tube, and the Bernoulli equation neglecting wall friction and acceleration forces was solved for solids concentration. This method was adopted by Magnusson *et al.* [33]. The third is to build a stable downward flow system with particle density deduced from mass flux of particles and measurement where phase velocities were nearly equal [18]. This method was widely used by researchers with appropriate modifications, like Matsuno *et al.* [19], Cutolo *et al.* [15], Lischer and Louge [20], Herbert *et al.* [21] and so on. In addition, liquid fluidized beds had been used by some investigators with the purpose to overcome the difficulty of obtaining stable suspensions of solids in gases, like Yamazaki *et al.* [25], Zhou *et al.* [26], Rundqvist *et al.* [10], and Liu *et al.* [22,31]. However, it is worthwhile to mention that the validity of such a calibration in gaseous suspensions is questionable due to the differences in the refractive index between gases and liquids [13], and the probes should be calibrated in the same medium for which they will be utilized. Different calibration methods used by researchers are summarized in Table 1.

The accuracy of solids concentration measurement by using an optical-fiber solids concentration probe is strongly dependent on the precision of the calibration technique utilized. The calibration of optical-fiber solids concentration probe in gas-solid environment is challenging due to the heterogeneity and instability of gas-solids flow. It is difficult to offer a series of standard gas-solid flow covering solids concentrations from 0 to packed bed. Because it is rather difficult to maintain a homogeneous gas-solids flow at high solids concentrations, the majority of calibration methods developed to date are focused on obtaining relatively homogeneous gas-solids flow at low solids concentrations. When applying them to the practical measurements, the calibration methods may be inaccurate or problematic. There are no widely-accepted calibration methods which cover a wide range of solids concentrations up to now. Thus, a feasible and simple method for the calibration of optical-fiber solids concentration probe which could solve the above mentioned questions of the existing calibration methods is urgently needed to be developed. More research on the calibration of optical-fiber solids concentration probe, especially experimental, is still required.

## 3. Experiment Setup and Calibration Procedure

**Figure 3.**
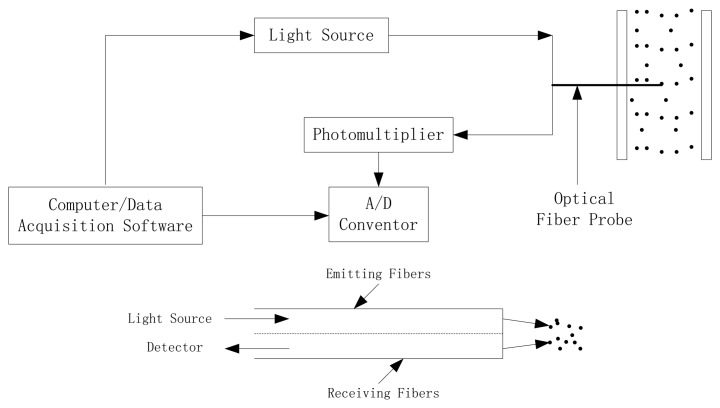
The optical-fiber probe system [16].

This study utilized a model PC6M optical-fiber solids concentration probe which was developed by the Institute of Process Engineering, Chinese Academy of Science, Beijing, China. This measurement system was composed of PC6M concentration measurement main unit, optical-fiber probes, and the signal cable, A/D converter and application software. The probe tip is 4 mm in diameter and contains approximately 8,000 emitting and receiving quartz fibers, each of a diameter of about 25 μm. These fibers are arranged in an alternating array, corresponding to emitting and receiving layers of fibers. The active area, where the fibers are located, is approximately 2 mm × 2 mm. The fiber tips are protected from the material by a glass window with thickness of 0.2 mm. The received light reflected by the particles is multiplied by the photo-multiplier and converted into a voltage signal. The voltage signal is further amplified and fed into a computer. The high voltage adjustment is used to adjust the upper measuring limit or full scale of the instrument. There is a zero voltage potentiometer which adjusts the output signal to zero when no powder is on the tip of the probe, thus the offsets of PC6M were set at zero with empty black box and the gains roughly at 4.5 V with packed box (less than the full range of 5 V), making the calibration procedure respond to most of possible particle concentrations. In order to make the day-to-day measurement comparable, the lamp voltage which sets the power voltage of the light source, and the gain factor should be kept constant during the calibration procedures [27]. Figure 3 shows the schematic diagram of the optical-fiber probe system.

**Figure 4.**
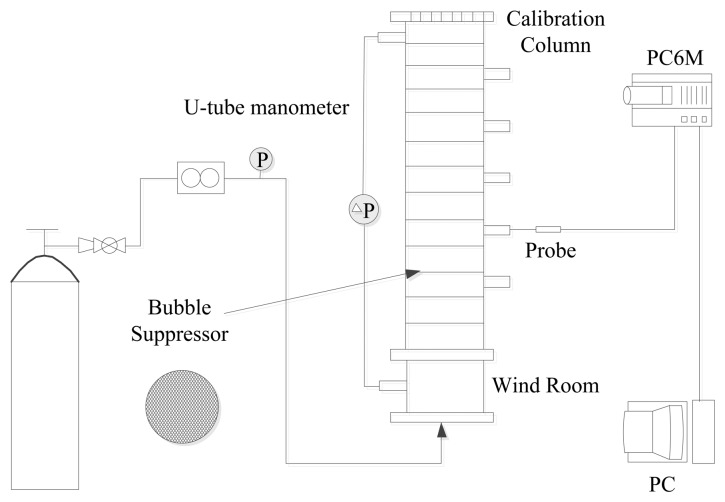
Calibration apparatus.

A Plexiglas column was used to calibrate the probes. As shown in Figure 4, the column is 50 mm in diameter and 300 mm high. A perforated Plexiglas plate covered with fine screens was employed as the gas distributor. Optical-fiber probes were installed along the column with their tips located on the axis of the column. Two pressure taps, one at the wind room and the other at the exit section of the column were provided to record the pressure drops. Pressure drop was measured by a 1 m long U-tube manometer, and water was used as the manometric fluid. The fluidizing gas was supplied by a nitrogen cylinder to the wind room which is 50 mm in diameter and 100 mm in length below the gas distributor. Bubble suppressor was installed in the calibration column, which contained a set of metal meshes with 25 mm axial pitches, the diameter of the metal mesh was 45 mm and the opening of each metal mesh was 3 mm × 3 mm with 0.5 mm steel wire. The top end of the column was covered with a filter screen to prevent the fine particles escaping from the column. Meanwhile, all metal portions of the whole calibration apparatus were carefully connected and grounded to eliminate static electricity effect.

**Figure 5.**
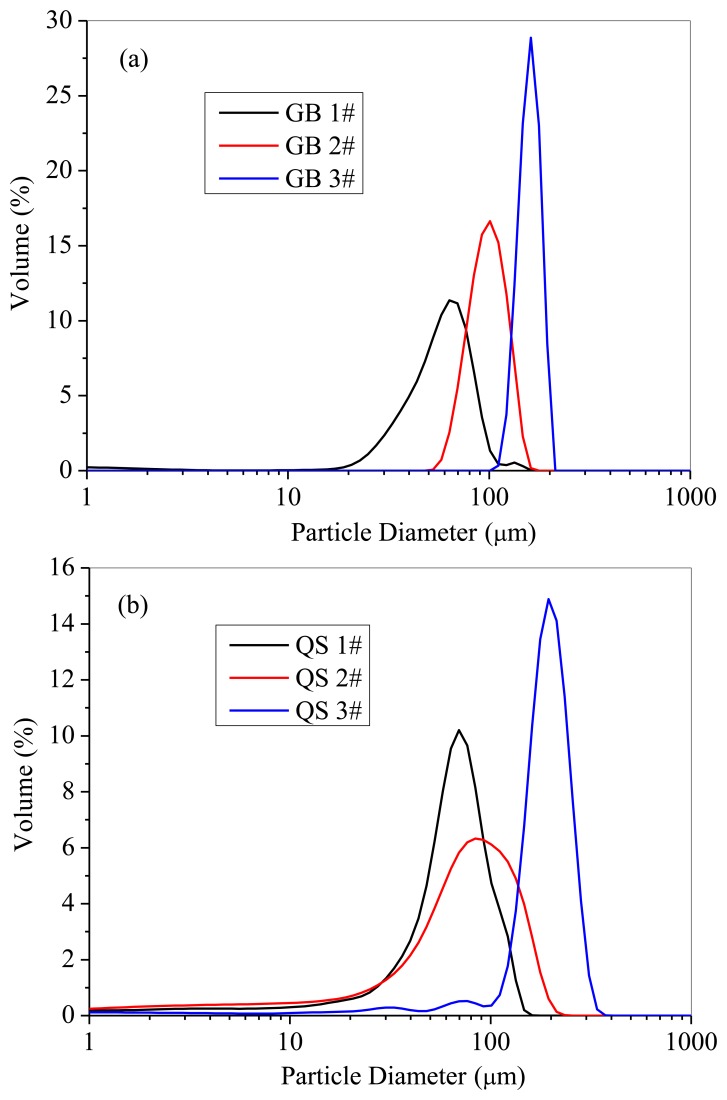
Particle size distribution: (**a**) Glass beads; (**b**) Quartz sand.

Glass beads and quartz sand were used to investigate the particle properties. Both of the two powders were sieved into three narrow distribution parts, and their physical properties are listed in Table 2. The particle size distributions were measured with a laser particle analyzer (LS, Beckman Coulter Inc., Brea, CA, USA), as shown in Figure 5. Because the particle diameter is much smaller than the bundle diameter, light is reflected by the particles in the measurement volume, allowing the probe to detect to measure the solids concentration.

The novel calibration procedure in the present study are consisted of two parts: one for high solids concentration (Method I) and the other for low solids concentration (Method II). The calibration procedure for high solids concentrations was similar to that of Qi [24] used a Pseudo Bubble-Free Fluidized Bed (PBFF). The powder was filled into the column with an initial bed height of about 100 mm, then fluidized by the fluidizing gas and was better distributed throughout the column with the assistance of bubble suppressors compared to without the installing of bubble suppressors.

During the calibration process, the optical-fiber probe was inserted into the column center between two successive bubble suppressor plates. The experiments were carried out under different ratio of *H*/*H_mf_*, where *H_mf_* is the bed height at minimum fluidization state and *H* is the bed height under other fluidizing gas flow rate. The solids concentration could be calculated according to the following equation:

(9) $$\frac{{ɛ}_{s}}{{ɛ}_{s,mf}}=\frac{H}{H_{mf}}$$

From the results obtained by this paper, this kind of calibration procedure was limited to solids concentration higher than 0.2. Thus, for the low solids concentration, the calibration data was obtained with the method similar to Hong *et al.* [23] and the detail procedures were with reference to Wang *et al.* [34]. All the calibration experiments were repeated 6 times under the same condition for the accuracy of the reproducibility. To minimize the influence of fine particles adhering to the tip surface of the probe on the calibration, particularly at the relatively high concentration, the optical-fiber probe was removed for cleaning of its surface for every test. In order to avoid the effect of any outside light source, the calibration column was covered with black cloth after fluidization of the solids particles. The sampling rate of the optical-fiber probe was 1 kHz and the sampling time approximately 4 s.

## 4. Results and Discussion

**Figure 6.**
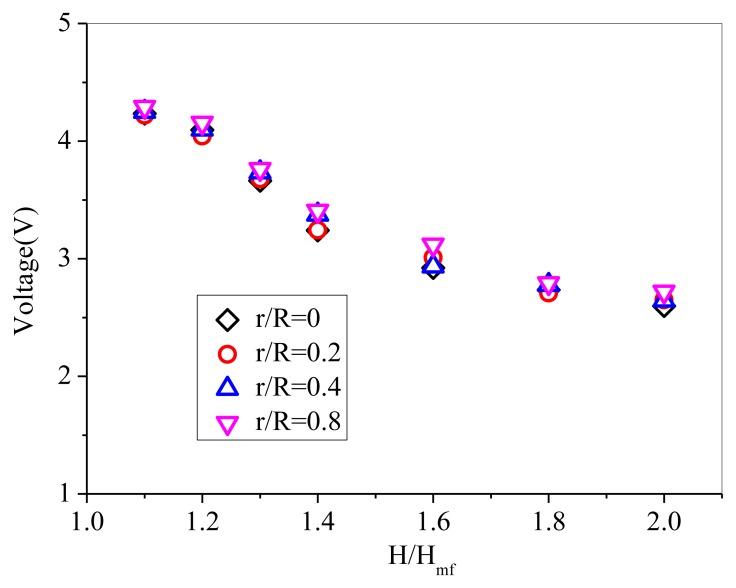
The probe output voltages of GB 3# at four different radial positions.

Due to that the complexity of the heterogeneous gas-solids flow manifests in the irregular, non-periodic variation of solids concentration with time, the typical time-resolved signals from the probe exhibit sharp spikes that correspond to the passage of individual particles in the near vicinity of the probe [20]. Different from pressure fluctuation signals, the solids concentration signals show binary behavior [35]. The peaks of the signals stand for high solids concentration and the valleys represent low solids concentration. During the calibration experiments, after reaching steady state, the mean output voltages of the probe were measured by immersing the probe at four radical positions: r/R = 0, r/R = 0.2, r/R = 0.4, r/R = 0.8. The radial voltage values of the probe are plotted in Figure 6. It can be seen that the output voltages of the probe at different radial positions basically agree well. The output voltages of the probe recorded near the wall is higher than those of the other three radial positions, which indicates higher solids concentration near the wall due to the wall effect. It can be inferred that relatively homogeneous suspension of solids was obtained.

In order to ensure that day-to-day measurements could be compared on an equal basis and to eliminate any parasitic effects caused by this particular apparatus which may prevent the use of the calibration curve in other systems, the output voltages were normalized according to the following expression [21]:

(10) $$v=\frac{V-V_{0}}{V_{p}-V_{0}}$$

where *V_0_* is the output voltage when the solids concentration is zero, and *V_p_* is the output voltage when the solids concentration is equal to packed bed concentration. According to the research of Cui *et al.* [30], the calibration correlations of the normalized output voltages and normalized solids concentration can be expressed as follows:

(11) $$\frac{{ɛ}_{s}}{{ɛ}_{s,p}}=\frac{av}{(1+a)-v}$$

where *ε_s,p_* is the packed bed solids concentration, *ε_s,p_*/*ε_s,p_* is normalized solids concentration, and *a* is the calibration coefficient.

**Figure 7.**
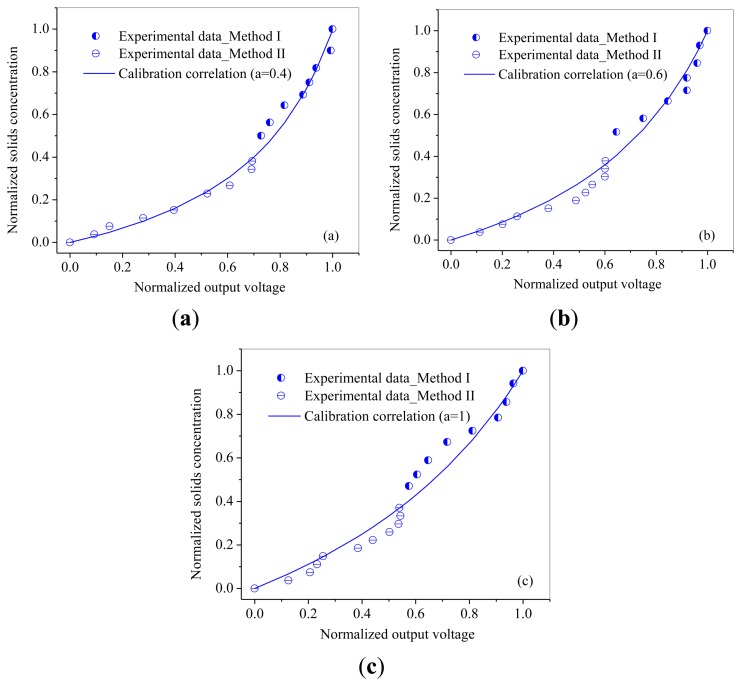
Calibration curves for Glass beads: (**a**) GB 1#; (**b**) GB 2#; (**c**) GB 3#.

**Figure 8.**
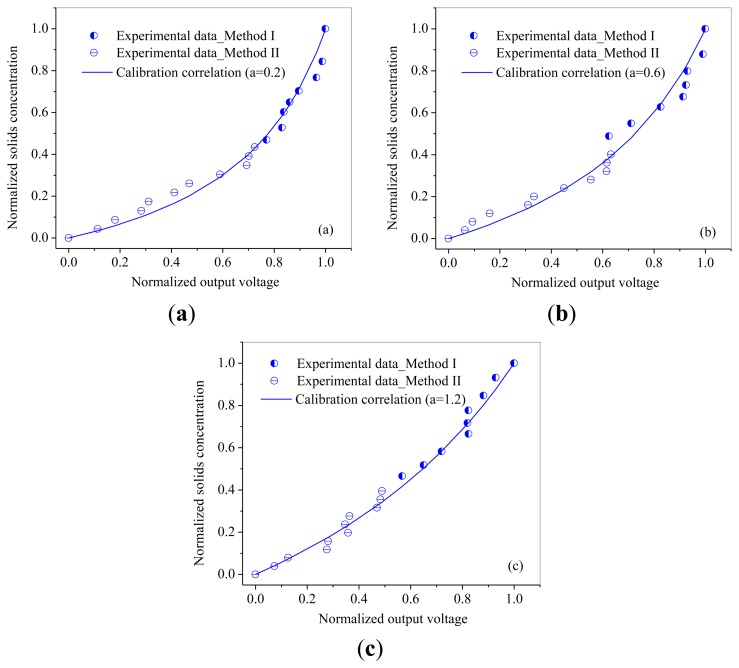
Calibration curves for Quartz sand: (**a**) QS 1#; (**b**) QS 2#; (**c**) QS 3#.

The relationships between normalized output voltages and normalized solids concentration are shown in Figures 7 and 8. From Figures 7 and 8, it can be seen that the output voltages increases sharply with increasing solids concentration at low solids concentrations, while increases slowly at high solids concentrations, which is consistent with the variation trend obtained by Cui *et al.* [30]. It may due to that for higher solids concentrations, the probe has a smaller measuring volume and lead to a more ‘local’ measurement because particles in the measuring volume woule be blocked by particles in front of them. Meanwhile, it can also been seen that the calibration coefficient *a* increases with the increasing of particle size.

Method II was used to calibrate the probe with a solids concentration range from 0.02 to 0.2 for both of the two powders. Method I worked at the solids concentrations from 0.26 to 0.53 for glass beads and from 0.22 to 0.5 for quartz sand. From Figures 7 and 8, it can also be seen that method I and method II together are capable of calibrating the optical-fiber probe at nearly full range of the solids concentrations from 0 to packed bed concentration.

**Figure 9.**
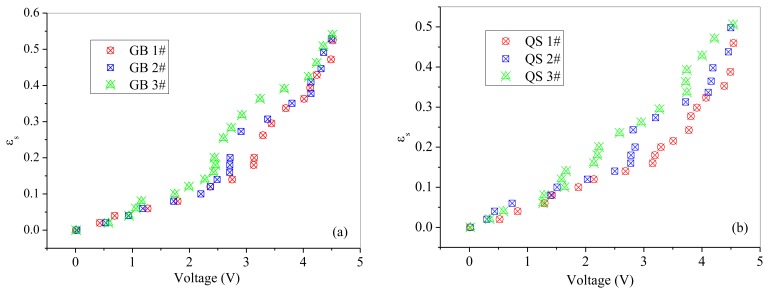
Effect of particle size on the calibration results: (**a**) Glass beads; (**b**) Quartz sand.

The suspensions encountered in practical problems of chemical engineering usually consist of particles with statistically varying irregular shapes, size and surface properties, locations and spatial orientations which are altogether unknown and for practical problems also may not be exactly describable [36]. The effect of particle size on calibration results is shown in Figure 9. It can be inferred that the output voltage of the optical fiber probe has a tendency to increase with decreasing particle size for the whole range of solid concentrations, but the increase tendency is not so obvious when the solids concentration less than 0.1. The intensity of the backscattered light is a function of the solids concentration and the mean particle size [25,37]. Yamazaki *et al.* [25] found that the intensity of the reflected light is affected by the particle diameter and the back-scattered light reach the probe decreases as the particle diameter increases. This may be attributed to the increase in the average path length of the light beam, which is caused by an increased particle diameter. Bos *et al.* [37] indicated that the intensity of the reflected light increases as the particle diameter decreases and the solids concentration increases. The present finding is consistent with their results. Meanwhile, Qi [24] also obtained similar conclusions that the output voltage decreases substantially with increasing particle size. According to Amos *et al.* [38], the relationship between the probe output and the solids concentration and the variation of this relationship seemed to be strongly dependent on the relationship between the particle size and the fiber diameter. With fixed fiber diameter, the relationship between the probe output and the solids concentration seemed to have the same slop at the low solids concentrations. In our study, the offset of the probe were adjusted to nearly zero with empty black box for each power with different particle diameter. Thus, the difference of calibration curves within the low solids concentration range between different particle sizes is not obvious. Amos *et al.* [38] also found that particle size effect was greater at higher solids concentrations, and that increasing the particle diameter while keeping constant the solids concentration and fiber diameter would lead to more light penetrate into the solid suspension. The light penetrating into the bed deeper than one particle would never be reflected out of the solids suspension. Meanwhile, the discrepancy of calibration results with different particle size exists at moderate solids concentration may also have some relationship with some heterogeneity effect in the gas-solid system due to that the inherent fluctuations are unable to be completely avoided in a gas-solid flow system. During the calibration experiments, it was found that the bubbles are more likely to form and expand at moderate solids concentrations. The bubble suppressor can reduce the generation of bubbles and avoid inherent fluctuations inside the calibration column to a certain extent, but it can't suppress the generation of bubbles totally. The degree of heterogeneity was relatively larger at moderate solids concentrations. Thus, a powder with very homogeneous fluidized behavior would be expected to have a more reliable calibration curve with this applied methodology.

**Figure 10.**
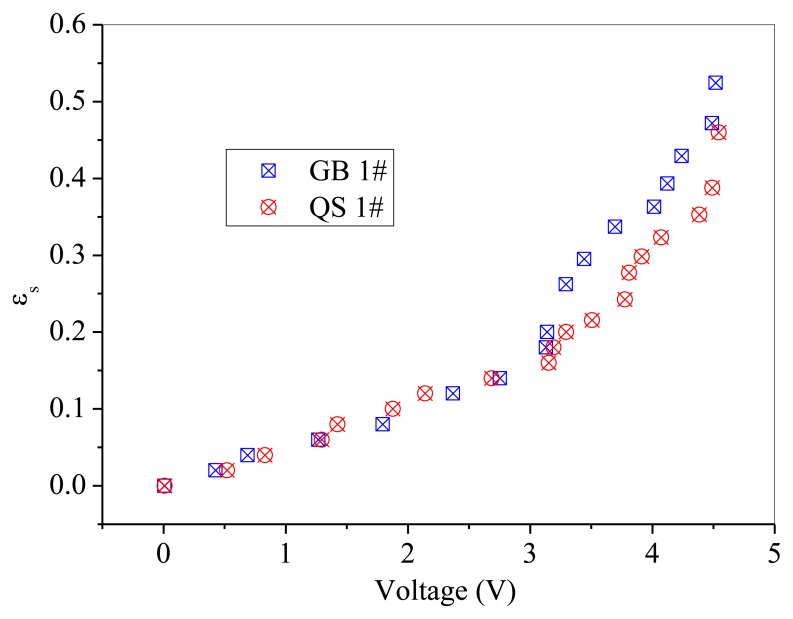
The compare of calibration results: GB1# and QS1#.

**Figure 11.**
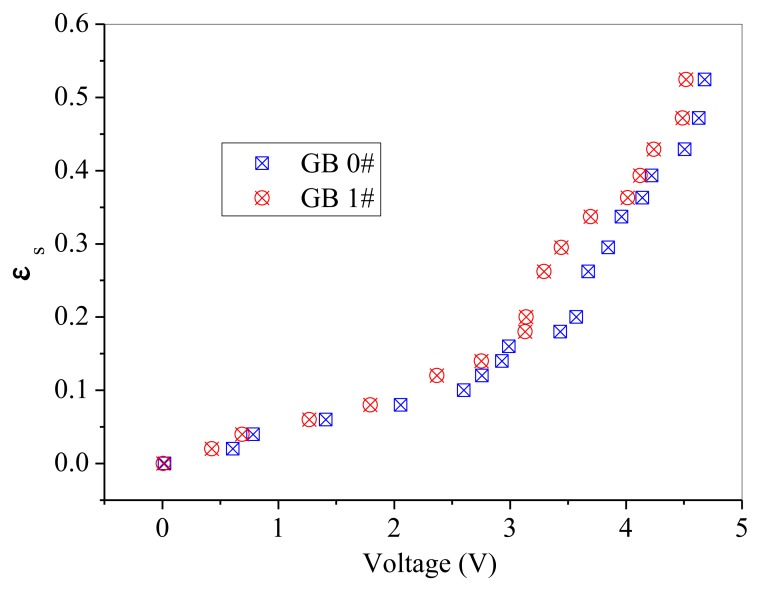
Effect of particle color on the calibration results.

**Figure 12.**
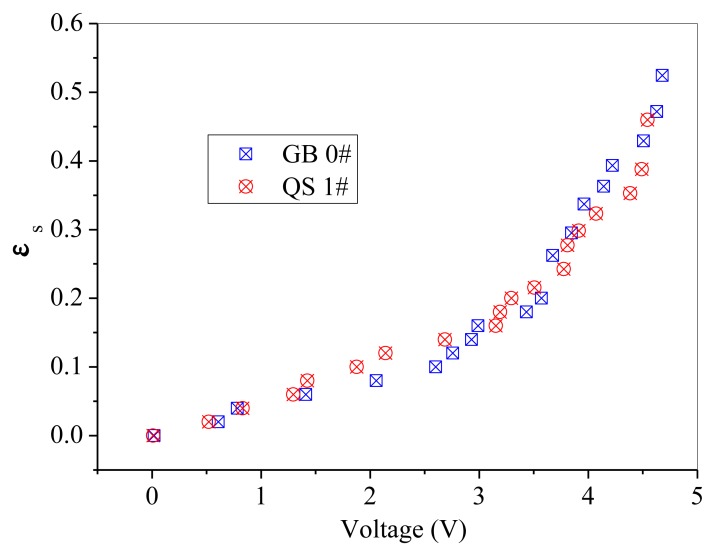
Effect of particle sphericity on the calibration results.

GB 1# and QS 1# are similar in particle size, but different in sphericity and color. Thus, comparisons are difficult to be made directly between these two powders, as shown in Figure 10. A batch of fresh glass beads with the same particle size as GB 1# but has a color of white, which was named GB 0#, was used to compare with the above mentioned two powders separately. The effect of particle color on calibration results is shown in Figure 11. The difference between GB 0# and GB 1# was color, and GB 1# was darker than GB 0#. It can be seen from Figure 11 that GB 0# has higher output voltages than those of GB 1#, which is due to that dark powder reflects less light [27]. The effect of particle sphericity on calibration results is shown in Figure 12. The calibration curves of GB 0# and QS 1# overlapped together. For sand particles, the sphericity is in the region of 0.8∼0.98 [10] which is usually less than that of glass beads. Rundqvist *et al.* [10] pointed out that the sampling light scattered from irregular particles will approximate the light scattered from the same number of spherical particles if a sufficient number of particles are considered, as the particles are oriented randomly in the suspension. The irregular object will appear more spherical as angular velocity increases, which is equivalent to sampling the same object from different angles. The impact of sphericity on the calibration theory was neglected in their research. Thus, it can be inferred that the effect of particle color on calibration result is more predominant than that of sphericity, Qi [24] also obtained the same conclusion. The calibration curves in Figure 10 are very close and overlapped at solids concentrations less than 0.2, which means that the calibration curve is less sensitive to the particle color when the solids concentration is low.

The difference of the calibration curve at solids concentration more than 0.2 may due to the color difference. The GB 1# are darker in color than QS 1#. Grey glass beads have the tendency to absorb more light compared to white quartz sand. It can be seen that under the same solids concentration, the output voltages of QS 1# are higher than that of GB 1#.

## 5. Conclusions

Different calibration methods of optical-fiber solids concentration probes reported in the literature were reviewed in this paper. Satisfactory calibration procedures are lacking in the literature, and the exact shape of the calibration function has not been verified exactly. A combined calibration method, which is capable of calibrating the optical-fiber probe at nearly full range of the solids concentrations from 0 to packed bed concentration, is proposed. With this combined method, the probe was uniquely calibrated with two kinds of powders. The effects of particle properties (particle size, sphericity and color) on the calibration results were comprehensively investigated. From the experiments carried out here, it can be concluded that the output voltage has a tendency to increase with the decreasing particle size, and the effect of particle color on the calibration curve is more predominant than that of sphericity.

## Figures and Tables

**Table 1.** Different calibration methods.

**Year**	**Author**	**Probe Type**	**Material**	**Particle Diameter**	**Particle Density**	**Calibration Apparatus**	**Verification Method**	**Linear/Non-Linear Calibration Curve**	**Remarks**	**Measuring System**
1983	Matsuno *et al.*	Reflection-type	Glass beads	56.5 μm	2520 kg/m^3^	vibrating sieves	Theoretical calculation	linear	Limited to low solids concentration	Gas-fluidized bed

1990	Cutolo *et al.*	Transmissi on-type	Glass beads	90 μm	-	A solids feed hopper with a 41 mm i.d. and 1 m high Plexiglas pipe	Theoretical calculation	linear	Good linearity when the volume was below 0.1	Highconcentration (up to 0.16) gas-solid suspension

1992	Lischer and Louge	Reflection-type	Glass beads	70 μm 210 μm	-	15 cm i.d. pipe	capacitance probe/simulatio n calculation	non-linear	Simple construction	-

1992	Yamazaki *et al.*	Reflection-type	Glass beads	225 μm	2,490 kg/m^3^	a flat-bottomed cylindrical tank of 6.0 cm diameter	Theoretical calculation	non-linear	The refractive index of liquids is different from that of gases	Slurry mixing tank
				131 μm	2,490 kg/m^3^					
				42 μm	2,350 kg/m^3^					
			Toyoura sands	163 μm	2,650 kg/m^3^					
			PVC powders	164 μm	1,500 kg/m^3^					

1994	Zhou *et al.*	Reflection-type	Ottawa sand	213μm	2,640 kg/m^3^	a liquid-solid fluidized bed/well-stirred water-sand beaker	Theoretical calculation	near-linear	The refractive index of liquids is different from that of gases	CFB of square cross-section

1994	Herbert *et al.*	Reflection-type	FCC particles	0.78 mm	1,630 kg/m^3^	A fluidized feeder with a 2.5 m long and 8 × 8 mm square cross-section tube	Theoretical calculation	non-linear	The volume fraction range calibrated was only 0.01 to 0.1	0.05 m diameter downflow CFB reactor

1994	Zhou *et al.*	Reflection-type	Ottawa sand	213 μm	2,640 kg/m^3^	a liquid-solid fluidized bed/well-stirred water-sand beaker	Theoretical calculation	near-linear	The refractive index of liquids is different from that of gases	CFB of square cross-section

1995	Hong *et al.*	Reflection-type	Limestone	0.124 mm	2,170 kg/m^3^	fluidized vessel	Theoretical calculation	non-linear	A powder with very homogeneous fluidized behavior is more suitable	Horizontal pneumatic piplies

1998	San José *et al.*	Reflection-type	Glass beads	3 mm	2,420 kg/m^3^	moving bed/60 mm i.d. column	image treatment system	linear	-	Conical spouted beds
				4 mm						
				5 mm						

1998	Zhang *et al.*	Reflection-type	FCC particles	49.4 μm	1,500 kg/m^3^	An incipiently fluidized bed and vibrating solids feeder with a 3.81 m downer	quick closing valves	non-linear	The solids concentration range calibrated could be from 0 to about 0.56	-
				59.0 μm	1,420 kg/m^3^					

2001	Johnsson *et al.*	Reflection-type	Silica sand	0.30 mm	-	cold CFB riser	Optical reference probe	non-linear	-	Electrically heated fluidized bed/CFB boiler

2001	Cui *et al.*	Reflection-type	FCC particles Sand	70 μm	1,673 kg/m^3^	Mixtures of FCC and amorphous transparent polystyrene	Theoretical calculation	non-linear	Use known solids concentration mixture to simulate gas-solid flow	Air-fluidized bed
				385 μm	2,650 kg/m^3^					

2003	Rundqvist *et al.*	Reflection-type	Silica sand	0.15 mm	-	small stirred tank with water	Theoretical calculation	near-linear	-	-
				0.20 mm						

2003	Liu *et al.*	Reflection-type	FCC particles	70 μm	-	3-D gas-solid suspension/well-mixed water-FCC tank	quick-closing valves/Theoretical calculation	non-linear	-	High-density CFB riser

2005	Magnusson *et al.*	Reflection-type	Silica sand	0.08 mm	2,600 kg/m^3^	circulating fluidized bed	pressure drop measurement	non-linear	-	CFB
				0.46 mm						

**Table 2.** Physical properties of powders.

**Powder**		**Average Diameter (μm)**	**Particle Poured Bulk Density (kg/m^3^)**	**Particle Density (kg/m^3^)**	**Color**
Glass beads	GB 1#	60.29	1,390	2,650	grey
	GB 2#	104.3	1,400		
	GB 3#	166.8	1,430		

Quartz sand	QS 1#	66.53	1,200	2,610	white
	QS 2#	78.85	1,300		
	QS 3#	192.8	1,320		
